# Overestimation and underestimation of youths’ health-related quality of life are associated with youth and caregiver positive screens for depression: results of a population-based study among youths with longstanding type 1 diabetes

**DOI:** 10.1186/s13098-022-00809-w

**Published:** 2022-03-09

**Authors:** Anna Stahl-Pehe, Silvia Selinski, Christina Bächle, Katty Castillo, Karin Lange, Reinhard W. Holl, Joachim Rosenbauer

**Affiliations:** 1 Institute for Biometrics and Epidemiology, German Diabetes Centre, Leibniz Centre for Diabetes Research, Auf’m Hennekamp 65, 40225 Düsseldorf, Germany; 2 German Centre for Diabetes Research (DZD), Munich-Neuherberg, Auf’m Hennekamp 65, 40225 Düsseldorf, Germany; 3 Medical Psychology Unit, Hannover Medical School, Hannover, Germany; 4 Institute of Epidemiology and Medical Biometry, ZIBMT, University of Ulm, Ulm, Germany

**Keywords:** Type 1 diabetes mellitus, Quality of life, Adolescents, Depression screening, Epidemiology, Psychology

## Abstract

**Background:**

This study aimed to analyze the extent and direction of disagreement between self- and proxy-reported quality of life (QoL) and the factors associated with QoL overestimation and underestimation by caregivers compared with self-reports.

**Methods:**

This study used data from population-based questionnaire surveys conducted in 2012–2013 and 2015–2016 with 11- to 17-year-olds with a duration of type 1 diabetes of 10 years or longer and their caregivers (n = 1058). QoL in youth was assessed via 10-item KIDSCREEN (KIDSCREEN-10) self- and proxy-reported questionnaires. The scores ranged from 0 to 100, with higher scores indicating better QoL. Depression screening was performed via the Center for Epidemiological Studies Depression Scale for Children for youths (CES-DC screen positive: score > 15) and WHO-5 Well-being Index for parents/caregivers (WHO-5 screen positive: score ≤ 50).

**Results:**

The mean self- and proxy-reported normalized KIDSCREEN-10 scores were 64.2 (standard deviation [SD] 11.4) and 66.1 (11.5), respectively. More caregivers overestimated (self-reported minus proxy-reported score < − 0.5*SD self-reported score) than underestimated (self-reported minus proxy-reported score > 0.5*SD self-reported score) youths’ QoL (37% versus 23%, p < 0.001). Youths who screened positive for depression (18%) were at higher risk of their QoL being overestimated and lower risk of their QoL being underestimated by caregivers than youths who screened negative for depression (RR_Overestimation_ 1.30 [95% CI 1.10–1.52], RR_Underestimation_ 0.27 [0.15–0.50]). Caregivers who screened positive for depression (28%) overestimated the QoL of their children less often and underestimated the QoL of their children more often than caregivers who screened negative for depression (RR_Overestimation_ 0.73 [0.60–0.89], RR_Underestimation_ 1.41 [1.14–1.75]).

**Conclusions:**

Caregivers often over- or underestimated their children’s QoL. Positive screens for depression among both youths and caregivers contributed to the observed differences between self- and caregiver-reported QoL.

**Supplementary Information:**

The online version contains supplementary material available at 10.1186/s13098-022-00809-w.

## Background

Health-related quality of life (QoL) is a well-established indicator and patient-reported outcome of the health of children and adolescents with chronic diseases, such as type 1 diabetes. It is a stated goal in international guidelines that diabetes care should result in a high level of QoL. Therefore, global guidelines recommend routine assessment of developmental progress in all domains of QoL [1]. Youths’ QoL can not only be reported by themselves but also assessed by others (e.g., caregivers). However, it is known that youths with a chronic disease and their parents tend to rate youths’ QoL differently, and these differences can make the interpretation of QoL measures difficult [2–5]. Disagreement between self and parents’ reports of youths’ QoL (informant discrepancy) does not necessarily imply that one set of data is less reliable than the other; rather, a disagreement could arise because parents and youths contribute different but valid information. However, informant discrepancy increases the need to identify variables that contribute to the observed differences in the perspectives of youths and their caregivers [2, 6]. It is important to determine how parents think about their children’s QoL because parental perceptions may affect disease management and the use of health services for their children [2, 4]. On the one hand, it is conceivable that caregivers overestimate youths’ QoL and, consequently, make no or too little effort to improve it. On the other hand, caregivers may underestimate youths’ QoL, resulting in additional worries and emotional stress. Identifying the factors behind the informant discrepancy may help in the interpretation of self- and proxy report QoL measures.

To date, few studies in youths with type 1 diabetes have addressed the possible causes of informant discrepancies concerning QoL. A study from Greece reported a significant difference between the QoL of youths with type 1 diabetes as reported by themselves and their parents. However, associated variables were not identified [3]. Among youths in the SEARCH study, larger discrepancies in parents’ and youths’ reports of QoL and parents’ underestimation were associated with youths’ higher hemoglobin A1_c_ (HbA1_c_) after adjusting for sociodemographic variables and diabetes duration [5]. Research from other collectives provides further and partially contradictory evidence on what may contribute to caregivers’ overestimation or underestimation of youths’ QoL. Factors under discussion to increase the discrepancies are the parent’s view of parent–child relationships, the children’s social support, the parental burden [7], the level of the child’s self-assessment, the parent’s assessment of the subjective health, reported emotional abnormalities [8], and the presence of depression in the child [9].

Depression is often comorbid with diabetes [10, 11] and is associated with decreased QoL [12–14]. That depression is associated with a decline in QoL is reasonable because depression is a mood disorder that affects how one feels, thinks, and behaves; therefore, it may promote a variety of emotional and physical problems. Currently, it is well known that depression is associated with factors important for the course and outcome of diabetes, including suboptimal glycemic control, incident micro- and macrovascular diseases, elevated mortality rates, and impaired QoL [10–14]. Current guidelines recommend depression screening for all youths with type 1 diabetes [1]. It has been estimated that the comorbidity of diabetes and depression in youths is a significant problem affecting approximately 20% of youths with diabetes. In addition, it has been reported that youths with type 1 diabetes for more than 10 years were more likely to be depressed than youths with shorter type 1 diabetes duration [10]. However, depression affects not only youths with type 1 diabetes themselves but is also common among their parents and impacts diabetes management and outcomes [15, 16]. It has been assumed that the mental health and well-being of caregivers may influence the reporting of their children’s QoL [17].

To the best of our knowledge, the research question of whether screens for depression in both youths with type 1 diabetes and caregivers can help explain differences between self- and caregiver-reported QoL has not been studied before. Therefore, the current study aims to (1) assess youths’ QoL according to self and caregiver reports and the resulting informant discrepancy and (2) analyze the relevance of depression screening results among youths and caregivers to explain parental over- and underestimation of youths’ QoL. We hypothesize that positive screens for depression are associated with increased discrepancies in self- and parent QoL ratings. We used existing data from a study population with early-onset type 1 diabetes for this evaluation. In addition to depression screening outcomes, sociodemographic and diabetes-related variables and further available data regarding other variables that have been found to explain informant discrepancies in other studies will be considered in the analyses.

## Participants and methods

### Data sources

The main data source was standardized self-administered questionnaires from the German nationwide population-based cohort study "Clinical Course of Type 1 Diabetes in Children, Adolescents and Young Adults with Disease Onset at Preschool Age". The study was approved by the ethics committee of Düsseldorf University (study number 3254). Details on the cohort study are given in Supplemental Figure S1 and described elsewhere [18, 19]. In short, the first baseline survey conducted from 2009–2010 was a questionnaire survey for youths with an onset of disease during their first five years of life who had a diabetes duration of at least ten years. The patients were selected from the anonymous nationwide diabetes register at the German Diabetes Center (DDZ). One of the DDZ register sources was the nationwide Diabetes-Prospective Follow-up registry (DPV) [20]. The treatment center or facility (hospital or medical practice) that reported the respective patient to the diabetes register forwarded the study documents to its patients. Additional baseline surveys were conducted with identical inclusion criteria 2012–2013 and 2015–2016 for youths with type 1 diabetes onset at a later date. Study participants who returned comprehensive questionnaires and informed consent to the study center were included in the baseline surveys and invited to follow-up surveys in 3-year intervals. Subjects who answered only the most important questions of short questionnaires for nonparticipants were not followed. When written consent was provided to the research group for linkage with clinical data on routine care procedures, the HbA1_c_ values documented in the pseudonymous DPV database were linked with the questionnaire data. For this investigation, only data from comprehensive questionnaires from the surveys conducted during 2012–2013 and 2015–2016 were used because data on the variables of interest were fully collected only in these surveys.

### Study population

Only participants younger than 18 years of age at the time of the survey were eligible for inclusion in this investigation. A total of 1163 11- to 17-year-olds fulfilled this inclusion criterion (N = 127 with type 1 diabetes onset from 1993 to 1999 and survey participation in 2012–2013, N = 452 with onset from 2000 to 2002 and survey participation in 2012–2013, and N = 584 with onset from 2003 to 2005 and survey participation in 2015–2016). An additional inclusion criterion was that the total score of the QoL measure KIDSCREEN-10 could be calculated. A total of 43 observations had to be excluded because of two or more missing values among youths. Additionally, 62 observations had to be excluded because the proxy report was incomplete. This resulted in a sample size of 1058 subjects (study group 1 (N = 115) with type 1 diabetes onset from 1993–1999 and survey participation in 2012–2013, study group 2 (N = 400) with onset from 2000–2002 and survey participation in 2012–2013, and study group 3 (N = 543) with onset from 2003–2005 and survey participation in 2015–2016).

### Variables

The youths and their caregivers answered the German versions of several internationally standardized and psychometrically validated screening instruments.

QoL was assessed using the generic short forms of the European KIDSCREEN questionnaire (KIDSCREEN-10) for children/adolescents (self-reports) and parents/caregivers (proxy reports). The KIDSCREEN-10 instrument is recommended for use in epidemiological surveys to assess subjective QoL in healthy and chronically ill children and adolescents. The KIDSCREEN-10 index is a singular index of global QoL and displays good psychometric properties [21–25]. The ten items of the KIDSCREEN-10 instrument cover the physical, psychological, and social aspects of QoL. The ratings were (re)coded such that higher values indicate better QoL; then, the item scores were summed and transformed into T-scores. The scoring algorithms were designed such that the mean T-value was 50 and the standard deviation was 10 for the entire European KIDSCREEN sample [21]. Thus, the given scoring algorithm for the KIDSCREEN-10 differs for the self- and proxy-reported versions and results in different minimum and maximum T-scores. Scoring algorithms are available for self-reports with one missing value but not for proxy reports with missing values [26]. To enable the comparison of self-reports with and without a missing value and proxy reports, we applied a min–max transformation to the self- and caregiver-reported T-scores to obtain normalized T-scores ranging from 0 to 100.

Youths were screened for depression by self-report using the Center for Epidemiologic Studies Depression Scale (CES-DC). The CES-DC was evaluated as a useful tool for epidemiologic studies of depression with psychometric properties acceptable for screening purposes [27–30]. The CES-DC total score was calculated by summing the unweighted 20-item scores, which resulted in a total score from 0 to 60. A higher total score indicates more pronounced depressive symptomatology. Scores higher than 15 are considered a positive screen for depression [27, 28, 31]. This published cutoff value was applied to define a binary CES-DC variable to differentiate participants screened positive or negative for depression.

Caregivers answered the World Health Organization-Five Well-Being Index (WHO-5), a questionnaire that measures current mental well-being and is recognized worldwide as a valid and reliable screening tool for depression [32, 33]. A sum score was calculated and transformed into a scale ranging from 0 (worst well-being) to 100 (optimal well-being). A total score ≤ 50 has been recommended as a cutoff value for a positive screen of depression [33–36]. The diagnostic accuracy of the WHO-5 for the screening of depression using a cutoff score of ≤ 50 is acceptable (weighted sensitivity was 0.86 and specificity was 0.81) [33]. We used this cutoff value for the definition of the binary WHO-5 variable and assigned the caregivers to the group with a positive or negative screen for depression.

Social support for children and adolescents was self-reported using the 8-item Social Support Scale (SSS-short). The items assess how frequently someone receives specific types of support in the form of listening, expressing affection, and providing problem-solving information when he or she needs it. A sum score was calculated and transformed on a scale ranging from 0 to 100, with higher scores indicating more available social support [29, 37, 38].

The socioeconomic status (SES) of the household, in which the youth was mainly living, was assessed using the Winkler Index for social strata according to the German Health Interview and Examination Survey for Children and Adolescents (KiGGS). This index integrates information given by caregivers about parental educational level, parental professional status, and household income. The three SES components of education, professional status, and household income were rated on a scale from 1 to 7; then, the component scores were summed, and youths were categorized into the following SES groups according to the caregiver with the highest SES: low (3–8 points), intermediate (9–14 points), and high (15–21 points) [39].

If an HbA1_c_ value locally measured within the period plus/minus 100 days to the completion date of the youth questionnaire was available in the DPV documentation, this value was selected (813 of 1058 participants). Otherwise, we used self- and caregiver-reported HbA1_c_ values (N = 232) to minimize missing values. For 132 participants, self- and proxy-reported HbA1_c_ values matched, 6 participants provided only HbA1_c_ self-reports, and only a proxy-reported HbA1_c_ was available for 54 participants. For 40 participants, self- and caregiver-reported data differed, with a mean difference (self-reported minus caregiver-reported HbA1c) of 0.35%. In these cases, the average of the self- and caregiver-reported HbA1_c_ was used. Before imputation, self- and proxy-reported HbA1_c_ values were set to missing (cf. statistical analyses).

Additional variables were study group, sex, age, household composition, caregiver who answered the proxy questionnaire, age at type 1 diabetes onset, diabetes duration, body mass index standard deviation score (BMI-SDS) based on German reference values [40, 41], and insulin pump therapy.

### Statistical analyses

Differences between self-reports and proxy reports were defined as self-reported minus caregiver-reported normalized KIDSCREEN-10 scores. Following other studies [4, 7, 8, 42] and the usual definition of a clinically meaningful difference in QoL [43], we determined agreement between the youth and caregiver QoL ratings when the calculated absolute difference was less than or equal to half a standard deviation (SD) of the self-reported score (in this sample, corresponding to a difference of ≤ 5.7 points). If there was no match, two types of disagreement were distinguished: underestimation when the proxy rating was lower/worse than the self-rating and overestimation when the proxy rating was higher/better than the self-rating.

Percentages or means and standard deviations (SDs) were calculated. Data for groups with parental over- and underestimation of youths’ QoL (over- and underestimation groups) were compared with data for the group with agreement between youth and caregiver QoL ratings (agreement group/reference group) in complete case analyses. Group comparisons of categorical and continuous variables were performed using chi-squared/Fisher’s exact tests (as appropriate) or Kruskal–Wallis tests, respectively.

Furthermore, we analyzed the association of a positive depression screening among youths and caregivers with parental over- or underestimation, each compared with the reference group in separate log-binomial regression models, adjusting for potentially influencing factors. The first model (Model 1) included only the two exposure variables of main interest—the binary CES-DC and WHO-5 depression screening variables—as independent variables. The second model (Model 2) was adjusted for sex, age, and diabetes duration. The third model (Model 3) was the result of the LASSO variable selection procedure [44] and additionally adjusted for the variables household composition, caregiver report, SES index, BMI-SDS, HbA1_c_, and SSS-short. In addition, we alternatively used the CES-DC and WHO-5 total scores as continuous independent exposure variables in Model 1.

We used Kendall’s *τ* and the corresponding test to study the pairwise relations of binary variables. In addition, we estimated the variance inflation factor for each variable to assess the potential multicollinearity effects. Both the pairwise correlations (all less than 0.5) and the estimated variance inflation factors (all less than 5) did not indicate any serious effects [45].

Because the proportion of missing values was up to 12% for the CES-DC covariate, the results of complete case analyses may be biased. Therefore, for a sensitivity analysis, we performed multiple imputations for missing data and created 30 versions of the original dataset. Before imputation, HbA1_c_ values based on self and proxy reports were set to missing. Missing data for continuous and categorical variables were replaced by imputed values generated using a fully conditional specification approach assuming “missing at random” [46, 47]. Then, log-binomial regression analyses were repeated using the multiple imputed data. Estimates of regression coefficients based on the 30 imputed datasets were summarized according to Rubin’s Rules and respective confidence intervals, and tests used small-sample-adjusted degrees of freedom [48]. Summarized relative risk estimates and respective confidence intervals were derived from the pooled estimates of regression coefficients.

The results of the log-binomial regression models are presented as relative risks (RRs) for overestimation and underestimation. Furthermore, we adjusted p-values for multiple-comparison testing per the Benjamini–Hochberg procedure to control for the false discovery rate (FDR) [49]. Two-sided p-values < 0.05 were considered statistically significant. We used RStudio (RStudio Team 2020), R version 3.6.2 and 4.0.0 (R Core Team 2020), and SAS version 9.4 (SAS Institute Inc., Cary, North Carolina, USA, 2016) for the analyses.

## Results

### Description of the study population

**Fig. 1:**
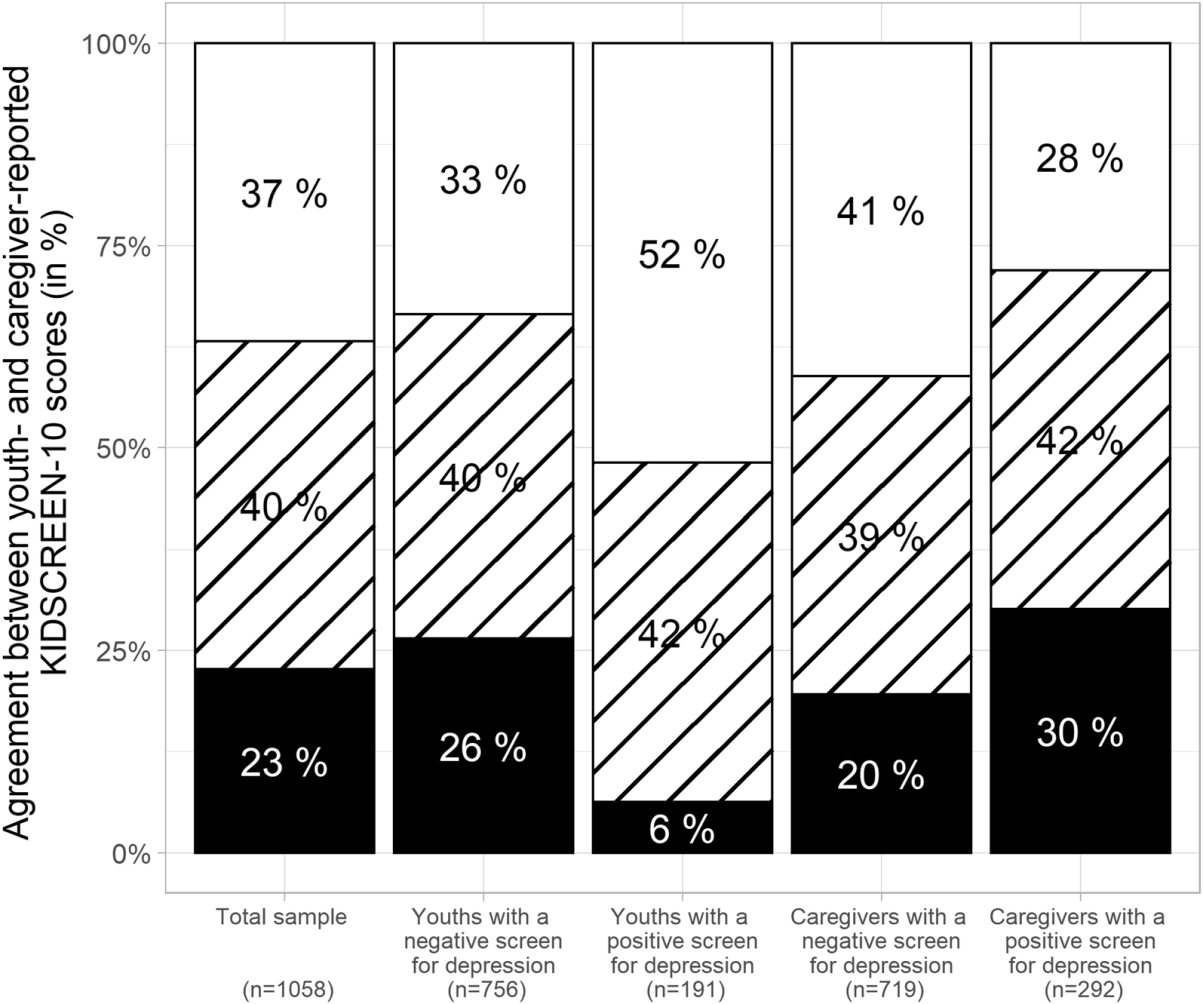
Agreement between youth- and caregiver-reported KIDSCREEN-10 scores differentiated according to the results of depression screening of the youths and caregivers. The percentage of youth–caregiver dyads in each group is given. The black color denotes underestimation, the diagonal lines indicate agreement, and the white color indicates overestimation of youth-reported QoL

A total of 515 girls and 543 boys with predominantly intermediate (45%) and high (42%) SES participated. The majority of the participating caregivers were mothers (72%). The mean age of the youths was 14.3 (SD 1.5) years, and the mean diabetes duration was 12.0 (1.2) years. The mean HbA1_c_ value was 8.2% (1.4%) (66.2 (15.3) mmol/mol). The mean self- and proxy-reported normalized KIDSCREEN-10 scores used to assess the youths’ QoL were similar (64.2 (11.4) and 66.1 (11.5) points, respectively) (Table 1). The study population characteristics stratified by the three study groups are given in Additional file 1: Table S1. More details on the normalized KIDSCREEN-10 scores are given in Additional file 1: Table S2. Agreement between self- and caregiver-reported QoL was found in 40% of all youth–caregiver dyads. More caregivers overestimated than underestimated youths’ QoL (37% versus 23%, p < 0.001) (Fig. 1).

**Table 1:** Characteristics of the study sample (total cohort and subgroups stratified based on the agreement of the self- and caregiver-KIDSCREEN-10 scores)

Characteristic	Total cohort		KIDSCREEN-10 ratings						p-value^d^	FDR adjusted p-value^e^
			Overestimation by caregivers^a^		Agreement of youth and caregiver ratings^b^		Underestimation by caregivers^c^			
	Percent or mean (SD)	n	Percent or mean (SD)	n	Percent or mean (SD)	n	Percent or mean (SD)	n		
Study group									0.6917	0.7246
1	11%	115	11%	44	11%	45	11%	26		
2	38%	400	39%	154	38%	164	34%	82		
3	51%	543	49%	192	51%	219	55%	132		
Sex									0.0037	0.0081
Boys	51%	543	47%	183	50%	215	60%	145		
Girls	49%	515	53%	207	50%	213	40%	95		
Age [years]	14.3 (1.5)	1058	14.4 (1.5)	390	14.4 (1.5)	428	14.14 (1.6)	240	0.0923	0.1562
Household composition									0.0661	0.1212
Biological parents	79%	828	80%	308	80%	343	74%	177		
Parent and partner	9%	92	6%	24	8%	35	14%	33		
Single parent	12%	123	13%	50	11%	45	12%	28		
Other	1%	11	1%	5	1%	4	1%	2		
Caregiver report by									0.5229	0.6391
Mother	72%	757	74%	289	68%	292	73%	176		
Father	8%	82	7%	27	8%	36	8%	19		
Mother and father	20%	212	18%	70	23%	98	18%	44		
Other	1%	6	1%	3	0%	2	0%	1		
SES index									0.7769	0.7769
Low SES	12%	123	11%	41	13%	56	11%	26		
Intermediate SES	45%	467	46%	177	44%	184	45%	106		
High SES	43%	450	43%	165	43%	179	45%	106		
Age at onset [years]	2.9 (1.2)	1058	3.0 (1.1)	390	2.9 (1.2)	428	2.7 (1.2)	240	0.0189	0.0378
Diabetes duration [years]	12.0 (1.2)	1058	11.9 (1.2)	390	12.0 (1.2)	428	11.9 (1.3)	240	0.6306	0.6937
BMI-SDS	0.3 (0.9)	1028	0.3 (0.9)	377	0.4 (1.0)	416	0.2 (0.9)	235	0.1880	0.2954
HbA1_c_ [mmol/mol]	66.2 (15.3)	1045	65.5 (14.7)	387	66.6 (14.7)	419	66.7 (17.0)	239	0.4509	0.5835
HbA1_c_ [%]	8.2 (1.4)	1045	8.2 (1.4)	387	8.2 (1.3)	419	8.3 (1.6)	239	0.4509	0.5835
< 7.5%	32%	338	36%	139	30%	126	31%	73	0.4241	0.5835
7.5–9.0%	47%	496	44%	172	49%	205	50%	119		
> 9.0%	20%	211	20%	76	21%	88	20%	47		
Insulin pump therapy									0.5888	0.6818
No	35%	372	35%	137	37%	156	33%	79		
Yes	65%	676	65%	251	63%	265	67%	160		
Social Support Scale (SSS-short)	83.8 (17.2)	994	80.8 (19.1)	361	83.6 (17.3)	404	88.9 (11.9)	229	< 0.0001	< 0.0002
KIDSCREEN-10 self	49.2 (10.1)	1058	45.0 (8.5)	390	48.5 (8.7)	428	57.3 (9.9)	240	< 0.0001	< 0.0002
KIDSCREEN-10 normalized self	64.2 (11.4)	1058	59.5 (9.6)	390	63.4 (9.9)	428	73.4 (11.2)	240	< 0.0001	< 0.0002
KIDSCREEN-10 caregiver	52.6 (12.0)	1058	59.6 (12.0)	390	50.0 (10.0)	428	46.0 (9.3)	240	< 0.0001	< 0.0002
KIDSCREEN-10 normalized caregiver	66.1 (11.5)	1058	72.7 (11.6)	390	63.5 (9.6)	428	59.7 (9.0)	240	< 0.0001	< 0.0002
CES-DC total score	10.2 (8.1)	929	12.2 (9.3)	344	10.2 (7.7)	374	6.8 (5.0)	211	< 0.0001	< 0.0002
Youth depression screening									0.0001	0.0002
Negative (CES-DC total score ≤ 15)	71%	756	65%	253	71%	303	83%	200		
Positive (CES-DC total score > 15)	18%	191	25%	99	19%	80	5%	12		
Unknown	10%	111	10%	38	11%	45	12%	28		
WHO-5 total score	57.9 (14.8)	1011	61.4 (14.4)	378	56.8 (14.2)	404	54.3 (15.3)	229	< 0.0001	< 0.0002
Caregiver depression screening									0.0001	0.0002
Negative (WHO-5 total score > 50)	68%	719	76%	296	66%	282	59%	141		
Positive (WHO-5 total score ≤ 50)	28%	292	21%	82	29%	122	37%	88		
Unknown	4%	47	3%	12	6%	24	5%	11		

^a^Difference between normalized self-reported minus caregiver-reported KIDSCREEN-10 score lower than half a standard deviation of the self-reported KIDSCREEN-10 score (difference < -0.5*SD)

^b^|Difference|≤ 0.5*SD

^c^Difference > 0.5*SD

^d^P-value of the chi-square test or Fisher’s exact test (as appropriate) in the case of categorical variables and of the Kruskal–Wallis test for quantitative variables testing for differences among the overestimation, agreement and underestimation groups

^e^P-value adjusted for multiple-comparison testing per the Benjamini–Hochberg procedure to control for the false discovery rate (FDR) [49]

### Depression screening results and QoL

Overall, half of the youth–caregiver dyads (51%) were screened negative for depression. Among youths, a total of 18% were screened positive for depression. Among caregivers, 28% were screened positive for depression. In 8% of the youth–caregiver dyads, both the youth and the caregiver were screened positive for depression (Table 2). Both concordant dyads with screen positivity or screen negativity for depression were more frequent than expected under the independence assumption (p_Fisher_ < 0.0001, *τ*_Kendall_ = 0.1694). Figure 1 shows that the proportion of caregivers who over- and underestimated youths’ QoL varied by youths’ and caregivers’ depression screening results. When the youths were screened positive for depression, their caregivers overestimated youths’ QoL more often (52% versus 33%) and underestimated it less often (6% versus 26%) than caregivers of youths who screened negative for depression (Fig. 1). Caregivers who screened positive for depression overestimated their children’s QoL less often (28% versus 41%) but underestimated it more often (30% versus 20%) than parents who screened negative for depression (Fig. 1).

**Table 2:** Results of depression screening among youths and caregivers (n (%))

Youth depression screening (CES-DC)	Caregiver depression screening (WHO-5)			
	Negative	Positive^a^	Unknown	Total
Negative	540 (51%)	182 (17%)	34 (3%)	756 (71%)
Positive^b^	100 (9%)	80 (8%)	11 (1%)	191 (18%)
Unknown	79 (7%)	30 (3%)	2 (0%)	111 (10%)
Total	719 (68%)	292 (28%)	47 (4%)	1058 (100%)

^a^WHO-5 total score ≤ 50

^b^CES-DC total score > 15

### Caregivers’ overestimation of youths’ QoL

In the group of participants with caregivers’ overestimation of youths’ QoL, the mean normalized KIDSCREEN caregiver-report score was 72.7, whereas the mean normalized KIDSCREEN self-report score was 59.5. The proportion of youths with a positive screen for depression was particularly high in this group (25%), and the proportion of caregivers with a positive screen for depression was lowest among the groups (21%) (Table 1).

Table 3 shows the relative risk of overestimation by caregivers associated with youth and caregiver depression screening results estimated from log-binomial regression models with increasing covariate adjustment. According to unadjusted Model 1, caregivers of youths who screened positive for depression were 30% more likely than caregivers of youths who screened negative for depression to overestimate the QoL of their children (RR 1.30 [95% CI 1.10–1.52]). After adjustment, the respective estimates were 26% according to Model 2 and 17% according to Model 3 (not statistically significant).

**Table 3:** Relative risks for caregiver overestimation and underestimation of normalized KIDSCREEN-10 scores compared with the reference group (Models 1–3 with binary depression screening variables)

Model	Variable		Overestimation versus agreement of youth and caregiver ratings				Underestimation versus agreement of youth and caregiver ratings			
			RR (95% CI)^a^	p-value^b^	FDR adjusted p-value^c^	N	RR (95% CI)^a^	p-value^b^	FDR adjusted p-value^c^	N
1	Youth depression screening (CES-DC)	Positive vs. negative	1.30 (1.10–1.52)	0.0017	0.0125	691	0.27 (0.15–0.50)	< 0.0001	< 0.0007	556
	Caregiver depression screening (WHO-5)	Positive vs. negative	0.73 (0.60–0.89)	0.0023	0.0125		1.41 (1.14–1.75)	0.0017	0.006	
2	Youth depression screening (CES-DC)	Positive vs. negative	1.26 (1.06–1.49)	0.0085	0.0340	691	0.28 (0.16–0.51)	< 0.0001	< 0.0007	556
	Caregiver depression screening (WHO-5)	Positive vs. negative	0.73 (0.60–0.90)	0.0025	0.0125		1.42 (1.15–1.77)	0.0018	0.0060	
	Sex	Girls vs. boys	1.07 (0.91–1.25)	0.4374	0.4860		0.96 (0.77–1.20)	0.7458	0.8287	
	Age [years]	Per one year increase	1.03 (0.96–1.01)	0.3916	0.4786		0.95 (0.86–1.04)	0.2728	0.3897	
	Duration [years]	Per one year increase	0.97 (0.89–1.05)	0.4068	0.4786		1.01 (0.89–1.14)	0.9351	0.9351	
3	Youth depression screening (CES-DC)	Positive vs. negative	1.17 (0.95–1.45)	0.1337	0.3343	626	0.38 (0.21–0.69)	0.0017	0.0060	519
	Caregiver depression screening (WHO-5)	Positive vs. negative	0.71 (0.57–0.88)	0.0021	0.0125		1.56 (1.25–1.95)	< 0.0001	< 0.0007	
	Sex	Girls vs. boys	1.15 (0.96–1.38)	0.1223	0.3343		0.91 (0.72–1.16)	0.4404	0.5505	
	Age [years]	Per one year increase	1.03 (0.96–1.11)	0.3518	0.4786		0.93 (0.85–1.02)	0.1405	0.2342	
	Duration [years]	Per one year increase	0.96 (0.87–1.04)	0.3081	0.4786		1.06 (0.94–1.19)	0.3585	0.4780	
	Household composition	Parent and partner vs. biological parents	^#^				1.46 (1.11–1.92)	0.0209	0.0523	
		Single parent vs. biological parents	^#^				1.00 (0.70–1.45)			
	Caregiver report by	Father vs. mother	0.91 (0.66–1.24)	0.2549	0.4786		1.00 (0.66–1.53)	0.5887	0.6926	
		Both parents vs. mother	0.84 (0.67–1.04)				0.86 (0.62–1.16)			
	SES index	Middle vs. low	1.21 (0.91–1.62)	0.3592	0.4786		^d^			
		High vs. low	1.13 (0.83–1.52)				^d^			
	BMI-SDS	1 unit	0.96 (0.91–1.04)	0.3979	0.4786		0.91 (0.81–1.03)	0.1227	0.2231	
	HbA1c [%]	1 unit	0.97 (0.91–1.04)	0.3981	0.4786		0.99 (0.90–1.09)	0.8956	0.9351	
	Social Support Scale (SSS-short)	10 units	0.95 (0.91–1.00)	0.0545	0.1817		1.15 (1.05–1.27)	0.0038	0.0109	

^a^RR: Relative risk with 95% confidence interval from the log-binomial model including all four independent variables

^b^P-value of the likelihood ratio test

^c^P-value adjusted for multiple-comparison testing per the Benjamini–Hochberg procedure to control for the false discovery rate (FDR) [49]

^d^Variable not selected by the Lasso method

In contrast, caregivers who screened positive for depression were 27% less likely to overestimate the QoL of their children than caregivers who screened negative for depression in Model 1 (RR 0.73 [0.60–0.89]). Respective estimates were 27% and 29%, according to Models 2 and 3, respectively. The other covariates in Model 3 were not statistically significantly associated with overestimation of youths’ QoL by caregivers.

The results of an analogous Model 1 performed with the CES-DC and WHO-5 total scores showed concordant associations in the same direction (Additional file 1: Table S3). Furthermore, the results from the multiple imputed dataset corroborated the findings from the original dataset with the exception that the youths’ depression screening variable was statistically significantly associated with QoL overestimation in all models (Additional file 1: Table S4).

### Caregivers’ underestimation of youths’ QoL

In the group of participants with caregivers’ underestimation of youths’ QoL, the mean normalized KIDSCREEN self-report score was 73.4, whereas the mean normalized KIDSCREEN caregiver-report score was 59.7. In this group, the proportion of youths who screened positive for depression was lowest (5%), whereas the proportion of parents who screened positive for depression was highest (37%) (Table 1).

The unadjusted log-binomial regression model revealed that caregivers of youths who screened positive for depression were 73% less likely than caregivers of youths who screened negative for depression to underestimate the QoL of their children (RR 0.27 [0.15–0.50]). The respective adjusted estimates from Models 2 and 3 were 72% and 62%, respectively. However, caregivers who screened positive for depression were 41% more likely than caregivers who screened negative for depression to underestimate their children’s QoL (RR 1.41 [1.14–1.75]) (Table 3). The respective adjusted estimates from Models 2 and 3 were 42% and 56%, respectively. In Model 3, the results suggested a potential association of a higher risk of caregivers’ underestimation of youths’ QoL with household composition and social support.

The CES-DC and WHO-5 total scores showed concordant associations of the same direction with caregivers’ underestimation of youths’ QoL (Additional file 1: Table S3). Furthermore, the results from the multiple imputed dataset corroborated the findings from the original dataset (Additional file 1: Table S4).

## Discussion

In this study, we were particularly interested in the associations between positive screens for depression and over- and underestimation of youths’ QoL. We gained insights that have not yet been reported from other samples. Both overestimation and underestimation of youths’ QoL were associated with youths’ and caregivers’ positive screens for depression.

Our results are mostly consistent with the findings of previous studies [16, 22, 28, 31, 50–52]. The mean self-reported KIDSCREEN-10 score was similar to those of other studies that used the KIDSCREEN-10 instrument (the European KIDSCREEN study and subsample with chronic health conditions [22]). However, the caregivers in our study rated youths’ QoL better than did parents in previous studies, despite the presumably more complex chronic health conditions of their children with type 1 diabetes. Notably, in contrast to the parents of the KIDSCREEN chronic health condition subsample, the caregivers in our study did not report worse youth QoL than the youths themselves [22]. We are aware of only one study in which the KIDSCREEN-10 index was collected from adolescents with type 1 diabetes. In comparison with this previous web-based study among 12- to 19-year-olds with shorter type 1 diabetes duration, we found slightly higher self-reported QoL in our sample. Caregiver-reported QoL was not assessed in that study [50]. The prevalence rates of positive screens for depression in youths and caregivers were lower than or similar to those reported in other studies. The mean CES-DC score and the proportion of youths with a positive screening for depression were just as high in our sample as in representative samples [28, 31]. This finding was in line with expectations based on previous studies [51, 52], although the proportion of youths who were screened positive for depression was comparatively low in our study [53]. The mean WHO-5 score of caregivers in our study was lower than that in the German reference population [34] and slightly lower than that among Dutch caregivers of 8- to 15-year-olds with a mean type 1 diabetes duration of 5 years. However, the proportion of caregivers with a positive screening for depression was similar [16]. We conclude that the youths and caregivers in our study reported common well-being and comparatively good QoL.

The tendency of the caregivers in our sample to rate their children’s QoL slightly better than the youths themselves is also reflected in the proportions of under- and overestimation within the paired QoL scores (23% underestimation and 37% overestimation). This result contrasts with that of previous studies that have reported that parents underestimate the QoL of their children more often than overestimate it, especially if a health care need is present [4, 54]. We assume that parents underestimated the effect that diabetes had on the lives of their children. Type 1 diabetes was a nearly lifelong experience for the youths in our study, and their caregivers were very experienced in dealing with the challenges of diabetes therapy after at least 10 years of illness. The growing independence of their children in therapy during adolescence and their children’s acceptable HbA1_c_ values might have promoted a positive image of their children. Our results suggest that findings from other groups, including those based on both representative samples and samples with other chronic diseases, cannot be easily transferred to youths with longstanding type 1 diabetes. Future studies should investigate whether the tendency of overestimation by caregivers is a particular feature of the sample studied or a typical finding in patients with early-onset type 1 diabetes treated with modern therapy options or broader populations living with type 1 diabetes.

We observed that caregivers who screened positive for depression were less likely than those who screened negative for depression to overestimate their children’s QoL. Moreover, they tended to underestimate their children’s QoL. Similar observations have been reported in other studies that parents with impaired well-being more often underestimate the QoL of their children [4, 42, 55]. This finding suggests that parents are guided in their judgment of the QoL of their children by their own feelings or project their emotions on their child’s disease [17, 54]. In addition, it has been observed that parents are often unaware of their child’s depressive symptoms [56]. However, because this study was not qualitative, we do not know how the differences can be explained. A qualitative study with 8- to 12-year-olds and their parents revealed that the children and parents based their answers to the KIDSCREEN-27 questionnaire on different experiences and different reasons [57]. We suspect the same causes in our study and assume that parents’ psychological condition affects their response behavior.

Our study is characterized by the strength of a nationwide population-based sample of children and adolescents with type 1 diabetes and their caregivers. In our opinion, the studied group of patients with early-onset, long-duration type 1 diabetes is particularly interesting and relevant because early-onset type 1 diabetes is an important determinant of long-term complications and survival [58]. Another strength lies in the standardized assessment of all variables using well-validated psychometric tools. All groupings were based on common practice cut-points to enable comparability. In addition, we considered sociodemographic and disease-related variables for adjustment in regression analyses.

This study was based on standardized, psychometrically tested screening instruments of a broad thematic spectrum to the extent that they were reasonable for young people. As is common practice in epidemiological studies, we relied on self-report questionnaires instead of structured diagnostic interviews to assess depression. Thus, we only screened for depression and had no evidence of clinically significant depression, which clearly represents a limitation of our study. This limitation also means that we cannot rule out that a positive screen for depression in this study might have been (subclinical) diabetes-specific emotional distress rather than psychopathology [59, 60].

We took into account several potentially confounding variables in regression analyses. However, we cannot exclude that additional variables for which data were not collected in this study, such as information on anxiety‐related disorders that are often comorbid with depression [61] or family functioning [62], were also associated with caregivers’ over- and underestimation of youths’ QoL. Contrary to our expectations based on the findings of the SEARCH study [5], the HbA1_c_ level was not associated with parental over- and underestimation of youths’ QoL. This can possibly be explained by the fact that HbA1c was locally measured in our study but centrally analyzed in the SEARCH study. The fact that we did not observe associations with sociodemographic covariates was in line with the findings from other studies [9, 63].

Another limitation is that this sample is not representative of all patients with type 1 diabetes and that nonresponses may have caused selection bias, limiting the generalizability of the study results. In this study, the proportion of participants with low SES was lower than in a representative study previously used for comparison [18]. However, an association of SES with over- and underestimation of youths’ QoL by caregivers was not observed (Tables 1 and 3). We observed no systematic differences between CES-DC and WHO-5 responders and nonresponders regarding sex, age, household composition, caregiver who answered the questionnaire, age at onset, and diabetes duration (Additional file 1: Tables S5a and S5b). CES-DC responders and nonresponders did not differ regarding KIDSCREEN self- and caregiver-report scores (Additional file 1: Table S5a). However, WHO-5 nonresponders reported lower KIDSCREEN self- and caregiver-report scores than WHO-5 responders (Additional file 1: Table S5b).

The categorization of the differences between youths and parents into three classes (overestimation, underestimation, and agreement) may have resulted in psychometric information being lost. However, categorization was necessary to be able to analyze predictors of relevant informant discrepancies and to achieve good interpretability of the results; notably, we based categorization on an established method for defining relevant differences [43]. The analytic approach selected (log-binomial models) has the advantage that relative risks could be estimated directly. The estimated parameters enable readers to assess the relevance of the depression screening results among youths and caregivers for parental over- and underestimation of youths’ QoL.

The findings from this study implicate that caregiver reports of youth QoL should not be treated as a substitute for self-reports because both over- and underestimation occur. Our findings add to the existing literature that the mental well-being of both the youth and the caregiver should be taken into account in the interpretation of self- and proxy-reported QoL. Thus, our research provides another argument for the preference of self-reported QoL while valuing caregiver reports as complementary information [4, 6, 64]. Particularly for youths with clinically relevant depressive symptoms representing an internalizing disorder, there is a risk that the youths’ QoL will be overestimated if only a parent report is provided. Thus, parents’ psychological condition should be taken into account when interpreting parents’ statements as part of a clinical study or medical consultation. This approach seems particularly appropriate when diabetes therapy decisions have to be made.

To conclude, this analysis provides some of the first insights into the associations between positive screens for depression in both youths with type 1 diabetes and their caregivers and the assessment of youths’ QoL. Positive screens for depression in both youths and caregivers contributed to the observed differences between self-reported and caregiver-reported QoL.

## Supplementary Information

**Additional file 1: Figure S1.** Overview cohort study. **Table S1. **Characteristics of study sample (total cohort and stratified for study groups). **Table S2. **Normalized youth- and caregiver-reported KIDSCREEN-10 scores. **Table S3. **Relative risks for caregiver overestimation and underestimation of normalized KIDSCREEN-10 scores associated with sex, age, CES-DC, and WHO-5 total scores. **Table S4. **Relative risks for caregiver overestimation and underestimation of normalized KIDSCREEN-10 scores compared with the reference group (Models 1-3 with binary depression screening variables) using multiple imputed dataset. **Table S5a. **Main characteristics of the study sample (total cohort, stratified for youth depression screening (CES-DC) and response analysis). **Table S5b. **Main characteristics of the study sample (total cohort, stratified by caregiver depression screening (WHO-5) and response analysis).

## Data Availability

The datasets used during the current study are available from the corresponding author on reasonable request.

## Abbreviations

QoL: Health-related quality of life
HbA1c: Hemoglobin A1c
KIDSCREEN-10: Generic short form of the European KIDSCREEN questionnaire
CES-DC: Center for Epidemiologic Studies Depression Scale
WHO-5: World Health Organization-Five Well-Being Index
SSS-short: 8-Item Social Support Scale
SES: Socioeconomic status
BMI-SDS: Body mass index standard deviation score
SD: Standard deviation
RR: Relative risk
