# Supplementary material for: Overestimation and underestimation of youths’ health-related quality of life are associated with youth and caregiver positive screens for depression: results of a population-based study among youths with longstanding type 1 diabetes
Source: Diabetol Metab Syndr. 2022 Mar 9;14:40. doi: 10.1186/s13098-022-00809-w (PMC8905804; doi:10.1186/s13098-022-00809-w)
Supplement: Supplementary file 1 — Additional file 1: Figure S1. Overview cohort study. Table S1. Characteristics of study sample (total cohort and stratified for study groups). Table S2. Normalized youth- and caregiver-reported KIDSCREEN-10 scores. Table S3. Relative risks for caregiver overestimation and underestimation of normalized KIDSCREEN-10 scores associated with sex, age, CES-DC, and WHO-5 total scores. Table S4. Relative risks for caregiver overestimation and underestimation of normalized KIDSCREEN-10 scores compared with the reference group (Models 1-3 with binary depression screening variables) using multiple imputed dataset. Table S5a. Main characteristics of the study sample (total cohort, stratified for youth depression screening (CES-DC) and response analysis). Table S5b. Main characteristics of the study sample (total cohort, stratified by caregiver depression screening (WHO-5) and response analysis). [file 13098_2022_809_MOESM1_ESM.docx]

**Overestimation and underestimation of youths’ health-related quality of life are associated with youth and caregiver positive screens for depression: Results of a population-based study among youths with longstanding type 1 diabetes**

**Online-Only Additional Material**

**Figure S1: Overview cohort study**

**
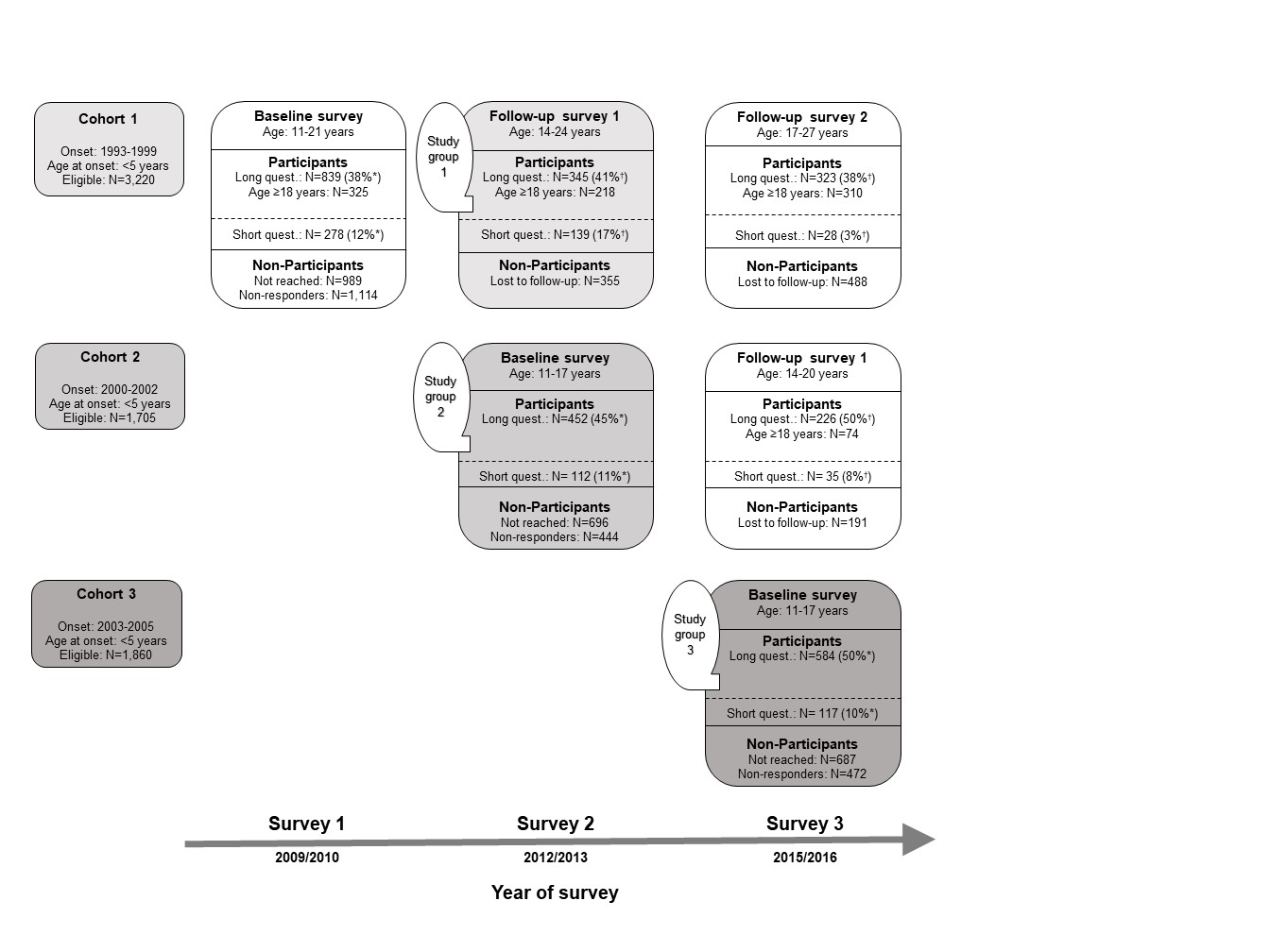
**

Long quest.: Study participants answered comprehensive questionnaires. Among 11- to 17-year-olds, parents answered additional comprehensive questionnaires.

Short quest.: Some participants answered only the most important questions of a short questionnaire.

* % of persons invited at baseline (eligible - not reached)

^†^ % of participants at baseline

**Table S1** Characteristics of study sample (total cohort and stratified for study groups)

|  | Total cohort | | Study group 1 | | Study group 2 | | Study group 3 | | p value^†^ |
| --- | --- | --- | --- | --- | --- | --- | --- | --- | --- |
| Characteristic | percent  or mean (SD) | n | percent  or mean (SD) | n | percent  or mean (SD) | n | percent  or mean (SD) | n |  |
| Study group |  |  |  |  |  |  |  |  |  |
| 1 | 11% | 115 |  |  |  |  |  |  |  |
| 2 | 38% | 400 |  |  |  |  |  |  |  |
| 3 | 51% | 543 |  |  |  |  |  |  |  |
| Sex |  |  |  |  |  |  |  |  |  |
| Boys | 51% | 543 | 52% | 60 | 49% | 196 | 53% | 287 | 0.4997 |
| Girls | 49% | 515 | 48% | 55 | 51% | 204 | 47% | 256 |  |
| Age [years] | 14.3 (1.5) | 1058 | 16.1 (1.0) | 115 | 14.2 (1.5) | 400 | 14.1 (1.2) | 543 | <0.0001 |
| Household composition |  |  |  |  |  |  |  |  |  |
| Biological parents | 79% | 828 | 81% | 93 | 82% | 330 | 75% | 405 | 0.0249 |
| Parent and partner | 9% | 92 | 7% | 8 | 6% | 23 | 11% | 61 |  |
| Single parent | 12% | 123 | 10% | 11 | 11% | 44 | 13% | 68 |  |
| Other | 1% | 11 | 3% | 3 | 1% | 3 | 1% | 5 |  |
| Caregiver report by |  |  |  |  |  |  |  |  |  |
| Mother | 72% | 757 | 78% | 90 | 71% | 282 | 71% | 385 | 0.5598 |
| Father | 8% | 82 | 6% | 7 | 9% | 34 | 8% | 41 |  |
| Mother and father | 20% | 212 | 15% | 17 | 21% | 82 | 21% | 113 |  |
| Other | 1% | 6 | 1% | 1 | 0% | 1 | 1% | 4 |  |
| SES index |  |  |  |  |  |  |  |  |  |
| Low SES | 12% | 123 | 17% | 19 | 12% | 49 | 10% | 55 | 0.3866 |
| Intermediate SES | 45% | 467 | 42% | 48 | 46% | 180 | 45% | 239 |  |
| High SES | 43% | 450 | 42% | 48 | 42% | 164 | 45% | 238 |  |
| Age at onset [years] | 2.9 (1.2) | 1058 | 2.3 (1.0) | 115 | 3.0 (1.1) | 400 | 3.0 (1.2) | 543 | <0.0001 |
| Diabetes duration [years] | 12.0 (1.2) | 1058 | 14.3 (0.9) | 115 | 11.7 (0.9) | 400 | 11.7 (0.9) | 543 | <0.0001 |
| BMI-SDS | 0.30 (0.93) | 1028 | 0.48 (0.97) | 113 | 0.25 (0.92) | 393 | 0.29 (0.92) | 522 | 0.0639 |
| HbA_1c_ [mmol/mol] | 66.2 (15.3) | 1045 | 66.5 (16.69) | 112 | 67.2 (15.9) | 396 | 65.4 (14.4) | 537 | 0.3556 |
| HbA_1c_ [%] | 8.2 (1.4) | 1045 | 8.2 (1.5) | 112 | 8.3 (1.5) | 396 | 8.1 (1.3) | 537 | 0.3556 |
| <7.5% | 32% | 338 | 36% | 40 | 31% | 124 | 32% | 174 | 0.0424 |
| 7.5-9.0% | 47% | 496 | 41% | 46 | 45% | 177 | 51% | 273 |  |
| >9.0% | 20% | 211 | 23% | 26 | 24% | 95 | 17% | 90 |  |
| Insulin pump therapy |  |  |  |  |  |  |  |  |  |
| No | 35% | 372 | 39% | 44 | 39% | 156 | 32% | 172 | 0.0426 |
| Yes | 65% | 676 | 61% | 70 | 61% | 239 | 68% | 367 |  |
| Social Support Scale (SSS-short) | 83.8 (17.2) | 994 | 83.3 (19.0) | 113 | 83.6 (17.3) | 378 | 84.1 (16.7) | 503 | 0.9144 |
| KIDSCREEN-10 self | 49.2 (10.1) | 1058 | 49.2 (10.7) | 115 | 48.5 (9.7) | 400 | 49.8 (10.1) | 543 | 0.0558 |
| KIDSCREEN-10 normalized self | 64.2 (11.4) | 1058 | 64.3 (12.1) | 115 | 63.4 (11.1) | 400 | 64.9 (11.4) | 543 | 0.0445 |
| KIDSCREEN-10 caregiver | 52.6 (12.0) | 1058 | 52.7 (11.4) | 115 | 52.2 (11.9) | 400 | 52.9 (12.2) | 543 | 0.8536 |
| KIDSCREEN-10  normalized caregiver | 66.1 (11.5) | 1058 | 66.2 (11.0) | 115 | 65.6 (11.5) | 400 | 66.3 (11.7) | 543 | 0.8536 |
| KIDSCREEN-10  normalized difference | -1.8 (11.7) | 1058 | -1.9 (11.8) | 115 | -2.3 (11.5) | 400 | -1.5 (11.9) | 543 | 0.2871 |
| CES-DC total score | 10.2 (8.1) | 929 | 9.5 (7.5) | 104 | 10.2 (7.6) | 345 | 10.3 (8.6) | 480 | 0.6852 |
| Youth depression screening (CES-DC) |  |  |  |  |  |  |  |  |  |
| Negative (total score ≤15) | 71% | 756 | 79% | 91 | 69% | 276 | 72% | 389 | 0.3386 |
| Positive (total score > 15) | 18% | 191 | 13% | 15 | 20% | 79 | 18% | 97 |  |
| Unknown | 10% | 111 | 8% | 9 | 11% | 45 | 10% | 57 |  |
| WHO-5 total score | 57.9 (14.8) | 1011 | 59.9 (15.8) | 110 | 58.4 (14.0) | 377 | 57.2 (15.1) | 524 | 0.1321 |
| Caregiver depression screening (WHO-5) |  |  |  |  |  |  |  |  |  |
| Negative (total score > 50) | 68% | 719 | 68% | 78 | 70% | 281 | 66% | 360 | 0.1682 |
| Positive (total score ≤ 50) | 28% | 292 | 28% | 32 | 24% | 96 | 30% | 164 |  |
| Unknown | 4% | 47 | 4% | 5 | 6% | 23 | 3% | 19 |  |
| ^†^ P value of the chi-square test or exact Fisher tests (as appropriate) in case of categorical variables and of the Kruskal-Wallis test in the case of quantitative variables testing for differences between the three study groups | | | | | | | | | |

**Table S2** Normalized youth- and caregiver-reported KIDSCREEN-10 scores

| T score | n | mean | SD | min | 25th quantile | median | 75th quantile | max |
| --- | --- | --- | --- | --- | --- | --- | --- | --- |
| Normalized youth report | 1058 | 64.2 | 11.4 | 35.9 | 56.3 | 63.3 | 69.6 | 100.00 |
| Normalized caregiver report | 1058 | 66.1 | 11.5 | 38.1 | 58.7 | 64.1 | 73.1 | 100.00 |
| Difference of normalized youth–caregiver couples ^†^ | 1058 | -1.8 | 11.7 | -43.7 | -8.9 | -2.1 | 4.9 | 42.90 |
| ^†^ Youth self-reported KIDSCREEN-10 score minus caregiver-reported score | | | | | | | | |

**Table S3** Relative risks for caregiver overestimation and underestimation of normalized KIDSCREEN-10 scores associated with sex, age, CES-DC, and WHO-5 total scores

|  |  | Overestimation versus agreement of youth and caregiver ratings (N=675) | | Underestimation versus agreement of youth and caregiver ratings (N=547) | |
| --- | --- | --- | --- | --- | --- |
| Variable |  | RR (95% CI) ^†^ | p value ^‡^ | RR (95% CI) ^†^ | p value ^‡^ |
| Youth CES-DC total score ^§^ | Per 10 units increase | 1.13 (1.08-1.18) | <0.0001 | 0.50 (0.40-0.63) | <0.0001 |
| Caregiver WHO-5 total score ^¶^ | Per 10 units increase | 0.87 (0.82-0.92) | <0.0001 | 1.17 (1.09-1.26) | <0.0001 |
| ^†^ RR: relative risk with 95% confidence interval  ‡ P value of the likelihood ratio test  ^§^ Higher CES-DC scores indicate a higher number of depressive symptoms.  ^¶^ Higher WHO-5 scores indicate better well-being. | | | | | |

**Table S4** Relative risks for caregiver overestimation and underestimation of normalized KIDSCREEN-10 scores compared with the reference group (Models 1-3 with binary depression screening variables) using multiple imputed dataset

|  |  |  | Overestimation versus agreement of youth and caregiver ratings | | Underestimation versus agreement of youth and caregiver ratings | |
| --- | --- | --- | --- | --- | --- | --- |
| Model | Variable |  | RR (95% CI) ^†^ | p value ^‡^ | RR (95% CI) ^†^ | p value ^‡^ |
| 1 | Youth depression screening (CES-DC) | Positive vs. negative | 1.29 (1.10-1.52) | 0.0020 | 0.36 (0.22-0.62) | 0.0002 |
|  | Caregiver depression screening (WHO-5) | Positive vs. negative | 0.76 (0.63-0.92) | 0.0049 | 1.36 (1.11-1.68) | 0.0032 |
| 2 | Youth depression screening (CES-DC) | Positive vs. negative | 1.28 (1.08-1.52) | 0.0043 | 0.39 (0.23-0.66) | 0.0005 |
|  | Caregiver depression screening (WHO-5) | Positive vs. negative | 0.76 (0.63-0.92) | 0.0048 | 11.39 (1.13-1.70) | 0.0019 |
|  | Sex | Girls vs. boys | 1.01 (0.87-1.18) | 0.8645 | 0.90 (0.73-1.10) | 0.3056 |
|  | Age [years] | Per one year increase | 1.01 (0.95-1.08) | 0.6449 | 0.91 (0.83-0.99) | 0.0320 |
|  | Duration [years] | Per one year increase | 0.97 (0.90-1.05) | 0.4718 | 1.07 (0.96-1.20) | 0.1961 |
| 3 | Youth depression screening (CES-DC) | Positive vs. negative | 1.22 (1.01-1.47) | 0.0385 | 0.47 (0.27-0.80) | 0.0056 |
|  | Caregiver depression screening (WHO-5) | Positive vs. negative | 0.76 (0.63-0.92) | 0.0046 | 1.45 (1.18-1.78) | 0.0004 |
|  | Sex | Girls vs. boys | 1.07 (0.92-1.25) | 0.4115 | 0.86 (0.69-1.06) | 0.1577 |
|  | Age [years] | Per one year increase | 1.02 (0.96-1.08) | 0.5654 | 0.91 (0.83-0.99) | 0.0315 |
|  | Duration [years] | Per one year increase | 0.97 (0.90-1.05) | 0.4369 | 1.10 (0.98-1.22) | 0.1112 |
|  | Household composition | Parent and partner vs. biological parents | ^§^ |  | 1.39 (1.07-1.81) | 0.0141 |
|  |  | Single parent vs. biological parents | ^§^ |  | 1.10 (0.81-1.49) | 0.5337 |
|  | Caregiver report by | Father vs. mother | 0.86 (0.64-1.16) | 0.3195 | 0.98 (0.68-1.42) | 0.9187 |
|  |  | Both parents vs. mother | 0.84 (0.69-1.02) | 0.0786 | 0.88 (0.67-1.15) | 0.3398 |
|  | SES index | Middle vs. low | 1.20 (0.92-1.55) | 0.1704 | ^§^ |  |
|  |  | High vs. low | 1.15 (0.88-1.50) | 0.2979 | ^§^ |  |
|  | BMI-SDS | Per 1 unit increase | 0.97 (0.90-1.06) | 0.5029 | 0.91 (0.82-1.02) | 0.0980 |
|  | HbA1_c_ [%] | Per 1 unit increase | 0.97(0.91-1.04) | 0.3627 | 1.02 (0.93-1.11) | 0.7029 |
|  | Social Support Scale (SSS-short) | Per 10 units increase | 0.96 (0.91 -1.00) | 0.0552 | 1.15 (1.06-1.25) | 0.0014 |
| ^†^ RR: Relative risk with 95% confidence interval from the log-binomial model including all four independent variables  ^‡^ P value of the likelihood ratio test  ^§^ Variable not selected by the Lasso-method | | | | | | |

**Table S5a** Main characteristics of the study sample (total cohort, stratified for youth depression screening (CES-DC) and response analysis)

|  | Total cohort | | Youths screened negative for depression  (CES-DC) | | Youths screened positive for depression (CES-DC) | | CES-DC responders | | CES-DC non responders | |  |
| --- | --- | --- | --- | --- | --- | --- | --- | --- | --- | --- | --- |
| Characteristic | percent or  mean (SD) | n | percent  or mean (SD) | n | percent  or mean (SD) | n | percent  or mean (SD) | n | percent  or mean (SD) | n | p value ^†^ |
| Sex |  |  |  |  |  |  |  |  |  |  | 0.3178 |
| Boys | 51% | 543 | 56.1 | 424 | 29.8 | 57 | 51% | 481 | 56% | 62 |  |
| Girls | 49% | 515 | 43.9 | 332 | 70.2 | 134 | 49% | 466 | 44% | 49 |  |
| Age [years] | 14.34 (1.53) | 1058 | 14.8 (1.53) | 756 | 15.06 (1.33) | 191 | 14.35 (1.53) | 947 | 14.24 (1.55) | 111 | 0.4625 |
| Household composition |  |  |  |  |  |  |  |  |  |  | 0.6302 |
| Biological parents | 79% | 828 | 79 | 595 | 74.9 | 143 | 78% | 738 | 82% | 90 |  |
| Parent and partner | 9% | 92 | 9.6 | 72 | 7.3 | 14 | 9% | 86 | 5% | 6 |  |
| Single parent | 12% | 123 | 10.4 | 78 | 16.8 | 32 | 12% | 110 | 12% | 13 |  |
| Other | 1% | 11 | 1.1 | 8 | 1 | 2 | 1% | 10 | 1% | 1 |  |
| Answer |  |  |  |  |  |  |  |  |  |  | 0.4555 |
| Mother | 72% | 757 | 71.9 | 543 | 73.3 | 140 | 72% | 683 | 67% | 74 |  |
| Father | 8% | 82 | 7.4 | 56 | 8.4 | 16 | 8% | 72 | 9% | 10 |  |
| Mother and father | 20% | 212 | 20.1 | 152 | 17.8 | 34 | 20% | 186 | 23% | 26 |  |
| Other | 1% | 6 | 0.5 | 4 | 0.5 | 1 | 1% | 5 | 1% | 1 |  |
| Age at onset [years] | 2.89 (1.15) | 1058 | 2.86 (1.14) | 756 | 3.07 (1.14) | 191 | 2.9 (1.15) | 947 | 2.82 (1.17) | 111 | 0.4832 |
| Diabetes duration [years] | 11.95 (1.21) | 1058 | 11.95 (1.23) | 756 | 11.99 (1.1) | 191 | 11.96 (1.2) | 947 | 11.89 (1.26) | 111 | 0.4447 |
| KIDSCREEN-10 self | 49.21 (10.05) | 1058 | 51.99 (8.83) | 756 | 37.36 (5.44) | 191 | 49.04 (10.13) | 947 | 50.72 (9.19) | 111 | 0.0904 |
| KIDSCREEN-10 normalized self | 64.23 (11.38) | 1058 | 67.37 (10.01) | 756 | 50.83 (6.15) | 191 | 64.03 (11.47) | 947 | 65.91 (10.43) | 111 | 0.0938 |
| KIDSCREEN-10 caregiver | 52.6 (11.98) | 1058 | 54.62 (11.62) | 756 | 43.97 (8.91) | 191 | 52.47 (11.91) | 947 | 53.7 (12.55) | 111 | 0.4190 |
| KIDSCREEN-10 normalized caregiver | 66.05 (11.53) | 1058 | 67.99 (11.18) | 756 | 57.76 (8.57) | 191 | 65.93 (11.46) | 947 | 67.12 (12.07) | 111 | 0.4190 |
| ^†^ P values were calculated using the exact Fisher test for 2x2 tables, the chi-squared test for kx2 tables, and the Wilcoxon/Kruskal-Wallis test for quantitative variables. P values >0.05 indicate that CES-DC nonresponders were comparable to responders regarding main characteristics. | | | | | | | | | | | |

**Table S5b** Main characteristics of the study sample (total cohort, stratified by caregiver depression screening (WHO-5) and response analysis)

|  | Total cohort | | Caregiver screened negative for depression (WHO-5) | | Caregiver screened positive for depression (WHO-5) | | WHO-5 responders | | WHO-5 non responders | |  |
| --- | --- | --- | --- | --- | --- | --- | --- | --- | --- | --- | --- |
| Characteristic | percent  or mean (SD) | n | percent  or mean (SD) | n | percent  or mean (SD) | n | percent  or mean (SD) | n | percent  or mean (SD) | n | p value ^†^ |
| Sex |  |  |  |  |  |  |  |  |  |  | 0.3735 |
| Boys | 51% | 543 | 52% | 374 | 51% | 148 | 52% | 522 | 45% | 21 |  |
| Girls | 49% | 515 | 48% | 345 | 49% | 144 | 48% | 489 | 55% | 26 |  |
| Age [years] | 14.34 (1.53) | 1058 | 14.35 (1.54) | 719 | 14.33 (1.51) | 292 | 14.35 (1.53) | 1011 | 14.17 (1.56) | 47 | 0.3931 |
| Household composition |  |  |  |  |  |  |  |  |  |  | 0.3796 |
| Biological parents | 79% | 828 | 81% | 578 | 73% | 214 | 79% | 792 | 78% | 36 |  |
| Parent and partner | 9% | 92 | 7% | 49 | 14% | 41 | 9% | 90 | 4% | 2 |  |
| Single parent | 12% | 123 | 11% | 81 | 12% | 35 | 12% | 116 | 15% | 7 |  |
| Other | 1% | 11 | 1% | 8 | 1% | 2 | 1% | 10 | 2% | 1 |  |
| Answer |  |  |  |  |  |  |  |  |  |  | 0.2377 |
| Mother | 72% | 757 | 71% | 512 | 73% | 213 | 72% | 725 | 68% | 32 |  |
| Father | 8% | 82 | 8% | 60 | 7% | 20 | 8% | 80 | 4% | 2 |  |
| Mother and father | 20% | 212 | 20% | 142 | 20% | 58 | 20% | 200 | 26% | 12 |  |
| Other | 1% | 6 | 1% | 5 | 0% | 0 | 0% | 5 | 2% | 1 |  |
| Age at onset [years] | 2.89 (1.15) | 1058 | 2.89 (1.15) | 719 | 2.93 (1.14) | 292 | 2.90 (1.15) | 1011 | 2.72 (1.23) | 47 | 0.3218 |
| Diabetes duration [years] | 11.95 (1.21) | 1058 | 11.96 (1.19) | 719 | 11.92 (1.27) | 292 | 11.95 (1.21) | 1011 | 11.97 (1.11) | 47 | 0.7104 |
| KIDSCREEN-10 self | 49.21 (10.05) | 1058 | 50.45 (10.03) | 719 | 46.72 (9.67) | 292 | 49.37 (10.06) | 1011 | 45.82 (9.07) | 47 | 0.0049 |
| KIDSCREEN-10 normalized self | 64.23 (11.38) | 1058 | 65.63 (11.36) | 719 | 61.41 (10.96) | 292 | 64.41 (11.4) | 1011 | 60.36 (10.33) | 47 | 0.0045 |
| KIDSCREEN-10 caregiver | 52.60 (11.98) | 1058 | 55.38 (11.85) | 719 | 46.69 (9.75) | 292 | 52.87 (11.95) | 1011 | 46.76 (11.33) | 47 | 0.0016 |
| KIDSCREEN-10 normalized caregiver | 66.05 (11.53) | 1058 | 68.73 (11.4) | 719 | 60.37 (9.38) | 292 | 66.32 (11.49) | 1011 | 60.44 (10.9) | 47 | 0.0016 |
| ^†^ P values were calculated using the exact Fisher test for 2x2 tables, the chi-squared test for kx2 tables, and the Wilcoxon/Kruskal-Wallis test for quantitative variables. P values >0.05 indicate that WHO-5 nonresponders were comparable to responders regarding main characteristics. | | | | | | | | | | | |
